# (25*R*)-5a-Spiro­stane-3,12-dione

**DOI:** 10.1107/S1600536808043687

**Published:** 2009-01-08

**Authors:** Tie-Ying Zi, Zhi-He Zang, Ming-Yong Yuan, Ling-Li Zheng

**Affiliations:** a The First Affiliated Hospital, Chengdu Medical College, Xindu 610500, People’s Republic of China; b Department of Pharmacy, Chengdu Medical College, Chengdu 610081, People’s Republic of China

## Abstract

The title compound, C_27_H_40_O_4_, was obtained from the oxidation of (25*R*)-3b-hydr­oxy-5a-spiro­stan-12-one (Hecogenin) by Jone’s reagent. The mol­ecule contains six alicyclic and heterocyclic rings, all *trans*-fused, among which four six-membered rings adopt similar chair conformations while two five-membered rings assume an envelope conformation.

## Related literature

For general background, see: Chakravarti *et al.* (1953 ▶); Djerassi *et al.* (1962 ▶); Huang *et al.* (2002 ▶).
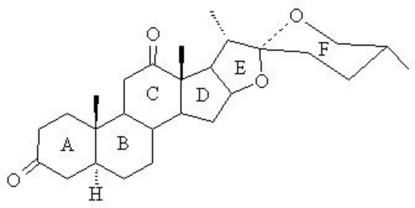


## Experimental

### 

#### Crystal data

C_27_H_40_O_4_
*M* *_r_* = 428.59
Monoclinic, 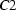

*a* = 12.660 (3) Å
*b* = 6.443 (3) Å
*c* = 30.167 (3) Å
β = 98.02 (3)°
*V* = 2436.8 (14) Å^3^
*Z* = 4
Mo *K*α radiation
μ = 0.08 mm^−1^
*T* = 292 (2) K
0.44 × 0.40 × 0.30 mm

#### Data collection

Enraf–Nonius CAD-4 diffractometer
Absorption correction: none
2837 measured reflections
2407 independent reflections
919 reflections with *I* > 2σ(*I*)
*R*_int_ = 0.136
3 standard reflections every 150 reflections intensity decay: 2.6%

#### Refinement

*R*[*F* ^2^ > 2σ(*F* ^2^)] = 0.078
*wR*(*F* ^2^) = 0.202
*S* = 1.07
2407 reflections
284 parameters
2 restraints
H-atom parameters constrained
Δρ_max_ = 0.19 e Å^−3^
Δρ_min_ = −0.23 e Å^−3^

### 

Data collection: *DIFRAC* (Gabe & White, 1993 ▶); cell refinement: *DIFRAC*; data reduction: *NRCVAX* (Gabe *et al.*, 1989 ▶); program(s) used to solve structure: *SHELXS97* (Sheldrick, 2008 ▶); program(s) used to refine structure: *SHELXL97* (Sheldrick, 2008 ▶); molecular graphics: *ORTEP-3 for Windows* (Farrugia, 1997 ▶); software used to prepare material for publication: *WinGX* (Farrugia, 1999 ▶).

## Supplementary Material

Crystal structure: contains datablocks I, global. DOI: 10.1107/S1600536808043687/xu2465sup1.cif

Structure factors: contains datablocks I. DOI: 10.1107/S1600536808043687/xu2465Isup2.hkl

Additional supplementary materials:  crystallographic information; 3D view; checkCIF report

## Figures and Tables

**Fig. 1.**
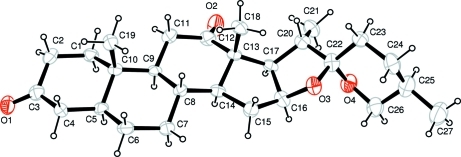
The molecular structure of the title compound.

## supplementary crystallographic information

### Comment

Steroid sapogenins are a class of important compounds, which present many significant bioactivities in platelet aggregation, arteriosclerosis and so on (Huang *et al.*, 2002). The hecogenin (25*R*-5a-Spirostane-3b-ol-12-one) is an important steroid (Djerassi *et al.*, 1962; Chakravarti *et al.*, 1953), which has been isolated from Chinese traditional medicine Tribulus terrestris. The title compound was recently obtained by Jone's oxidation of hecogenin in our laboratory, and its crystal structure is presented here.

The molecular structure of the title compound is shown in Fig. 1. The molecule contains six alicylic and heterocyclic rings with *trans*-fused. Cyclohexane rings A (C1/C2/C3/C4/C5/C10), B (C5/C6/C7/C8/C9/C10), C (C8/C9/C11/C12/C13/C14) and six-membered heterocyclic ring F (C22/C23/C24/C25/C26/O4) adopt chair conformation. Cyclopetane ring D (C13/C14/C15/C16/C17) and five-membered heterocyclic ring E (C16/C17/C20/C22/O3) have envelope conformation.

### Experimental

A solution of hecogenin (200 mg) in acetone (10 ml) was added by Jone's reagent drops by drops. The reaction solution was stirred for 20 min and quenched by water (10 ml). The mixture was extracted with CH_2_Cl_2_ (5 ml×3) and the organic phase was concentrated to give the title compound. The crystals suitable for X-ray analysis were obtained by slow evaporation from acetone solution at room temperature. The absolute configuration was deduced not by the present experiment but by the synthetic path from Hecognin, whose absolute configuration is known.

### Refinement

H atoms were located geometrically with C—H = 0.93–0.98 Å and refined using a riding model with *U*_iso_(H) = 1.2U_eq_(C). Friedel pairs were merged and the absolute configuration was not determined.

### Crystal data

**Table:**

C_27_H_40_O_4_	*F*(000) = 936
*M**_r_* = 428.59	*D*_x_ = 1.168 Mg m^−^^3^
Monoclinic, *C*2	Mo *K*α radiation, λ = 0.71073 Å
Hall symbol: C 2y	Cell parameters from 12 reflections
*a* = 12.660 (3) Å	θ = 4.3–5.6°
*b* = 6.443 (3) Å	µ = 0.08 mm^−^^1^
*c* = 30.167 (3) Å	*T* = 292 K
β = 98.02 (3)°	Block, colourless
*V* = 2436.8 (14) Å^3^	0.44 × 0.40 × 0.30 mm
*Z* = 4	

### Data collection

**Table:**

Enraf–Nonius CAD-4 diffractometer	*R*_int_ = 0.136
Radiation source: fine-focus sealed tube	θ_max_ = 25.5°, θ_min_ = 1.4°
graphite	*h* = −15→15
ω/2θ scans	*k* = −7→7
2837 measured reflections	*l* = −35→36
2407 independent reflections	3 standard reflections every 150 reflections
919 reflections with *I* > 2σ(*I*)	intensity decay: 2.6%

### Refinement

**Table:**

Refinement on *F*^2^	Primary atom site location: structure-invariant direct methods
Least-squares matrix: full	Secondary atom site location: difference Fourier map
*R*[*F*^2^ > 2σ(*F*^2^)] = 0.078	Hydrogen site location: inferred from neighbouring sites
*wR*(*F*^2^) = 0.202	H-atom parameters constrained
*S* = 1.07	*w* = 1/[σ^2^(*F*_o_^2^) + (0.0752*P*)^2^] where *P* = (*F*_o_^2^ + 2*F*_c_^2^)/3
2407 reflections	(Δ/σ)_max_ < 0.001
284 parameters	Δρ_max_ = 0.19 e Å^−^^3^
2 restraints	Δρ_min_ = −0.23 e Å^−^^3^

### Special details

**Table:**

Geometry. All esds (except the esd in the dihedral angle between two l.s. planes) are estimated using the full covariance matrix. The cell esds are taken into account individually in the estimation of esds in distances, angles and torsion angles; correlations between esds in cell parameters are only used when they are defined by crystal symmetry. An approximate (isotropic) treatment of cell esds is used for estimating esds involving l.s. planes.
Refinement. Refinement of *F*^2^ against ALL reflections. The weighted *R*-factor *wR* and goodness of fit *S* are based on *F*^2^, conventional *R*-factors *R* are based on *F*, with *F* set to zero for negative *F*^2^. The threshold expression of *F*^2^ > σ(*F*^2^) is used only for calculating *R*-factors(gt) *etc*. and is not relevant to the choice of reflections for refinement. *R*-factors based on *F*^2^ are statistically about twice as large as those based on *F*, and *R*- factors based on ALL data will be even larger.

### Fractional atomic coordinates and isotropic or equivalent isotropic displacement parameters (Å^2^)

**Table:**

	*x*	*y*	*z*	*U*_iso_*/*U*_eq_	
O1	0.7481 (5)	0.2933 (11)	0.4779 (2)	0.0958 (19)	
O2	0.6375 (4)	0.7105 (13)	0.7368 (2)	0.117 (3)	
O3	1.0031 (4)	0.7234 (9)	0.83970 (17)	0.0791 (17)	
O4	0.9410 (5)	1.0093 (10)	0.8752 (2)	0.096 (2)	
C1	0.6736 (5)	0.3818 (15)	0.5873 (2)	0.076 (2)	
H1A	0.6156	0.3421	0.6035	0.091*	
H1B	0.6664	0.5291	0.5809	0.091*	
C2	0.6615 (6)	0.2652 (16)	0.5435 (3)	0.086 (2)	
H2A	0.5996	0.3182	0.5243	0.104*	
H2B	0.6485	0.1200	0.5493	0.104*	
C3	0.7588 (7)	0.2814 (15)	0.5186 (4)	0.079 (2)	
C4	0.8630 (6)	0.2756 (16)	0.5476 (2)	0.080 (2)	
H4A	0.9174	0.3246	0.5304	0.096*	
H4B	0.8795	0.1325	0.5557	0.096*	
C5	0.8694 (5)	0.4009 (12)	0.5893 (2)	0.0635 (19)	
H5	0.8585	0.5464	0.5804	0.076*	
C6	0.9812 (5)	0.3854 (15)	0.6177 (2)	0.081 (2)	
H6A	1.0353	0.4246	0.5995	0.097*	
H6B	0.9944	0.2429	0.6273	0.097*	
C7	0.9890 (5)	0.5280 (16)	0.6591 (2)	0.076 (3)	
H7A	1.0577	0.5077	0.6773	0.092*	
H7B	0.9848	0.6717	0.6494	0.092*	
C8	0.9014 (5)	0.4848 (13)	0.6870 (2)	0.0585 (19)	
H8	0.9136	0.3454	0.6996	0.070*	
C9	0.7921 (5)	0.4852 (11)	0.6594 (2)	0.0541 (18)	
H9	0.7852	0.6255	0.6468	0.065*	
C10	0.7822 (6)	0.3447 (10)	0.6186 (3)	0.056 (2)	
C11	0.7016 (5)	0.4700 (13)	0.6871 (2)	0.075 (2)	
H11A	0.6966	0.3275	0.6970	0.089*	
H11B	0.6354	0.5032	0.6681	0.089*	
C12	0.7124 (7)	0.6099 (15)	0.7276 (3)	0.081 (2)	
C13	0.8200 (5)	0.5999 (11)	0.7571 (2)	0.0549 (19)	
C14	0.9030 (5)	0.6332 (12)	0.7254 (2)	0.0607 (19)	
H14	0.8864	0.7692	0.7116	0.073*	
C15	1.0041 (6)	0.6679 (15)	0.7598 (3)	0.088 (3)	
H15A	1.0313	0.5373	0.7727	0.105*	
H15B	1.0596	0.7357	0.7459	0.105*	
C16	0.9656 (6)	0.8067 (14)	0.7951 (2)	0.076 (2)	
H16	0.9878	0.9511	0.7922	0.091*	
C17	0.8411 (5)	0.7863 (14)	0.7912 (2)	0.065 (2)	
H17	0.8073	0.9126	0.7778	0.078*	
C18	0.8278 (6)	0.3872 (12)	0.7799 (2)	0.077 (2)	
H18A	0.8814	0.3915	0.8058	0.115*	
H18B	0.8467	0.2845	0.7594	0.115*	
H18C	0.7602	0.3523	0.7889	0.115*	
C19	0.7859 (7)	0.1141 (13)	0.6329 (3)	0.076 (2)	
H19A	0.7276	0.0855	0.6492	0.114*	
H19B	0.8521	0.0863	0.6516	0.114*	
H19C	0.7801	0.0275	0.6068	0.114*	
C20	0.8194 (6)	0.7661 (15)	0.8389 (2)	0.076 (2)	
H20	0.7965	0.6227	0.8428	0.091*	
C21	0.7318 (6)	0.9049 (17)	0.8505 (3)	0.098 (3)	
H21A	0.7527	1.0474	0.8483	0.146*	
H21B	0.7190	0.8765	0.8805	0.146*	
H21C	0.6678	0.8796	0.8301	0.146*	
C22	0.9283 (7)	0.7917 (16)	0.8671 (3)	0.084 (3)	
C23	0.9441 (6)	0.6702 (13)	0.9110 (3)	0.078 (2)	
H23A	0.9425	0.5224	0.9048	0.093*	
H23B	0.8867	0.7017	0.9282	0.093*	
C24	1.0524 (8)	0.729 (2)	0.9385 (3)	0.118 (4)	
H24A	1.0583	0.6638	0.9678	0.141*	
H24B	1.1100	0.6768	0.9235	0.141*	
C25	1.0628 (8)	0.956 (2)	0.9439 (4)	0.123 (4)	
H25	1.0063	1.0032	0.9607	0.147*	
C26	1.0440 (8)	1.0586 (18)	0.8978 (3)	0.109 (3)	
H26A	1.0507	1.2080	0.9012	0.130*	
H26B	1.0977	1.0116	0.8801	0.130*	
C27	1.1729 (7)	1.030 (3)	0.9702 (3)	0.164 (5)	
H27A	1.2271	1.0205	0.9510	0.247*	
H27B	1.1917	0.9440	0.9960	0.247*	
H27C	1.1668	1.1718	0.9795	0.247*	

### Atomic displacement parameters (Å^2^)

**Table:**

	*U*^11^	*U*^22^	*U*^33^	*U*^12^	*U*^13^	*U*^23^
O1	0.146 (5)	0.071 (4)	0.068 (4)	−0.012 (4)	0.005 (3)	−0.003 (4)
O2	0.076 (4)	0.156 (7)	0.117 (4)	0.044 (4)	0.003 (3)	−0.043 (5)
O3	0.081 (4)	0.082 (4)	0.074 (3)	0.026 (3)	0.009 (3)	−0.004 (3)
O4	0.105 (5)	0.079 (5)	0.101 (4)	0.006 (4)	0.000 (4)	−0.005 (4)
C1	0.064 (5)	0.082 (6)	0.087 (5)	−0.003 (5)	0.029 (4)	−0.009 (5)
C2	0.092 (6)	0.066 (6)	0.098 (6)	−0.015 (5)	0.001 (5)	−0.013 (6)
C3	0.098 (7)	0.051 (5)	0.087 (7)	−0.001 (5)	0.011 (6)	−0.002 (6)
C4	0.092 (6)	0.085 (6)	0.068 (5)	−0.020 (6)	0.029 (4)	−0.013 (6)
C5	0.066 (5)	0.054 (5)	0.072 (5)	−0.007 (4)	0.014 (4)	0.003 (4)
C6	0.074 (6)	0.073 (6)	0.102 (6)	−0.015 (4)	0.037 (5)	−0.012 (6)
C7	0.054 (4)	0.097 (7)	0.077 (5)	−0.005 (5)	0.010 (4)	−0.003 (5)
C8	0.049 (4)	0.055 (5)	0.072 (5)	0.000 (4)	0.013 (4)	−0.009 (4)
C9	0.062 (4)	0.043 (4)	0.059 (4)	−0.008 (4)	0.014 (4)	0.015 (4)
C10	0.059 (5)	0.036 (4)	0.075 (5)	0.006 (3)	0.019 (4)	0.004 (4)
C11	0.065 (5)	0.066 (6)	0.091 (6)	−0.001 (4)	0.007 (4)	−0.009 (5)
C12	0.068 (6)	0.082 (6)	0.097 (6)	0.007 (5)	0.028 (5)	−0.013 (6)
C13	0.053 (4)	0.046 (4)	0.068 (5)	0.004 (4)	0.018 (4)	0.013 (4)
C14	0.065 (5)	0.047 (4)	0.071 (5)	0.008 (4)	0.015 (4)	0.024 (4)
C15	0.063 (5)	0.113 (8)	0.089 (6)	−0.002 (5)	0.018 (5)	−0.024 (6)
C16	0.101 (7)	0.056 (5)	0.074 (5)	0.021 (5)	0.021 (5)	0.008 (4)
C17	0.071 (5)	0.063 (5)	0.064 (5)	0.005 (5)	0.018 (4)	0.025 (4)
C18	0.110 (6)	0.050 (4)	0.076 (5)	0.004 (5)	0.032 (5)	0.014 (4)
C19	0.107 (6)	0.047 (5)	0.075 (5)	0.001 (5)	0.015 (5)	0.002 (4)
C20	0.086 (6)	0.059 (5)	0.084 (6)	0.007 (5)	0.016 (5)	0.006 (5)
C21	0.104 (7)	0.108 (7)	0.084 (6)	0.019 (6)	0.028 (5)	−0.010 (6)
C22	0.102 (7)	0.063 (6)	0.083 (6)	0.023 (6)	0.005 (6)	−0.005 (6)
C23	0.086 (6)	0.067 (6)	0.081 (5)	0.004 (5)	0.014 (5)	0.013 (5)
C24	0.127 (9)	0.143 (13)	0.079 (7)	0.019 (8)	−0.002 (6)	0.019 (7)
C25	0.105 (8)	0.142 (13)	0.114 (8)	0.007 (8)	−0.007 (7)	−0.026 (7)
C26	0.132 (9)	0.093 (9)	0.102 (6)	0.002 (7)	0.021 (7)	−0.028 (6)
C27	0.129 (9)	0.221 (16)	0.133 (8)	−0.024 (11)	−0.015 (7)	−0.036 (11)

### Geometric parameters (Å, °)

**Table:**

O1—C3	1.220 (8)	C13—C18	1.530 (10)
O2—C12	1.211 (9)	C13—C17	1.579 (11)
O3—C22	1.412 (9)	C14—C15	1.547 (9)
O3—C16	1.464 (8)	C14—H14	0.9800
O4—C26	1.421 (9)	C15—C16	1.523 (10)
O4—C22	1.428 (12)	C15—H15A	0.9700
C1—C2	1.509 (10)	C15—H15B	0.9700
C1—C10	1.574 (9)	C16—C17	1.569 (9)
C1—H1A	0.9700	C16—H16	0.9800
C1—H1B	0.9700	C17—C20	1.509 (10)
C2—C3	1.533 (11)	C17—H17	0.9800
C2—H2A	0.9700	C18—H18A	0.9600
C2—H2B	0.9700	C18—H18B	0.9600
C3—C4	1.478 (10)	C18—H18C	0.9600
C4—C5	1.488 (10)	C19—H19A	0.9600
C4—H4A	0.9700	C19—H19B	0.9600
C4—H4B	0.9700	C19—H19C	0.9600
C5—C6	1.552 (9)	C20—C21	1.504 (10)
C5—C10	1.549 (9)	C20—C22	1.524 (10)
C5—H5	0.9800	C20—H20	0.9800
C6—C7	1.543 (11)	C21—H21A	0.9600
C6—H6A	0.9700	C21—H21B	0.9600
C6—H6B	0.9700	C21—H21C	0.9600
C7—C8	1.508 (9)	C22—C23	1.529 (11)
C7—H7A	0.9700	C23—C24	1.546 (12)
C7—H7B	0.9700	C23—H23A	0.9700
C8—C14	1.500 (9)	C23—H23B	0.9700
C8—C9	1.511 (8)	C24—C25	1.478 (17)
C8—H8	0.9800	C24—H24A	0.9700
C9—C11	1.512 (9)	C24—H24B	0.9700
C9—C10	1.521 (9)	C25—C26	1.529 (14)
C9—H9	0.9800	C25—C27	1.579 (13)
C10—C19	1.546 (11)	C25—H25	0.9800
C11—C12	1.510 (10)	C26—H26A	0.9700
C11—H11A	0.9700	C26—H26B	0.9700
C11—H11B	0.9700	C27—H27A	0.9600
C12—C13	1.521 (10)	C27—H27B	0.9600
C13—C14	1.533 (9)	C27—H27C	0.9600
			
C22—O3—C16	105.3 (6)	C15—C14—H14	105.3
C26—O4—C22	112.1 (7)	C16—C15—C14	103.9 (6)
C2—C1—C10	115.0 (6)	C16—C15—H15A	111.0
C2—C1—H1A	108.5	C14—C15—H15A	111.0
C10—C1—H1A	108.5	C16—C15—H15B	111.0
C2—C1—H1B	108.5	C14—C15—H15B	111.0
C10—C1—H1B	108.5	H15A—C15—H15B	109.0
H1A—C1—H1B	107.5	O3—C16—C15	109.5 (7)
C1—C2—C3	113.9 (7)	O3—C16—C17	103.5 (6)
C1—C2—H2A	108.8	C15—C16—C17	108.3 (6)
C3—C2—H2A	108.8	O3—C16—H16	111.7
C1—C2—H2B	108.8	C15—C16—H16	111.7
C3—C2—H2B	108.8	C17—C16—H16	111.8
H2A—C2—H2B	107.7	C20—C17—C16	104.5 (5)
O1—C3—C4	124.2 (8)	C20—C17—C13	121.2 (7)
O1—C3—C2	120.9 (8)	C16—C17—C13	100.9 (6)
C4—C3—C2	114.9 (8)	C20—C17—H17	109.8
C3—C4—C5	115.2 (7)	C16—C17—H17	109.8
C3—C4—H4A	108.5	C13—C17—H17	109.8
C5—C4—H4A	108.5	C13—C18—H18A	109.5
C3—C4—H4B	108.5	C13—C18—H18B	109.5
C5—C4—H4B	108.5	H18A—C18—H18B	109.5
H4A—C4—H4B	107.5	C13—C18—H18C	109.5
C4—C5—C6	111.7 (6)	H18A—C18—H18C	109.5
C4—C5—C10	113.4 (6)	H18B—C18—H18C	109.5
C6—C5—C10	109.7 (6)	C10—C19—H19A	109.5
C4—C5—H5	107.3	C10—C19—H19B	109.5
C6—C5—H5	107.3	H19A—C19—H19B	109.5
C10—C5—H5	107.3	C10—C19—H19C	109.5
C5—C6—C7	111.1 (6)	H19A—C19—H19C	109.5
C5—C6—H6A	109.4	H19B—C19—H19C	109.5
C7—C6—H6A	109.4	C21—C20—C17	114.2 (7)
C5—C6—H6B	109.4	C21—C20—C22	116.4 (8)
C7—C6—H6B	109.4	C17—C20—C22	104.6 (6)
H6A—C6—H6B	108.0	C21—C20—H20	107.1
C8—C7—C6	111.8 (7)	C17—C20—H20	107.1
C8—C7—H7A	109.3	C22—C20—H20	107.1
C6—C7—H7A	109.3	C20—C21—H21A	109.5
C8—C7—H7B	109.3	C20—C21—H21B	109.5
C6—C7—H7B	109.3	H21A—C21—H21B	109.5
H7A—C7—H7B	107.9	C20—C21—H21C	109.5
C14—C8—C7	112.4 (6)	H21A—C21—H21C	109.5
C14—C8—C9	109.6 (6)	H21B—C21—H21C	109.5
C7—C8—C9	112.5 (6)	O3—C22—O4	109.7 (8)
C14—C8—H8	107.4	O3—C22—C20	105.6 (7)
C7—C8—H8	107.4	O4—C22—C20	105.8 (7)
C9—C8—H8	107.4	O3—C22—C23	109.3 (7)
C8—C9—C11	113.8 (5)	O4—C22—C23	110.9 (8)
C8—C9—C10	114.2 (6)	C20—C22—C23	115.3 (8)
C11—C9—C10	115.2 (6)	C22—C23—C24	109.8 (7)
C8—C9—H9	103.9	C22—C23—H23A	109.7
C11—C9—H9	103.9	C24—C23—H23A	109.7
C10—C9—H9	103.9	C22—C23—H23B	109.7
C9—C10—C5	109.8 (5)	C24—C23—H23B	109.7
C9—C10—C19	110.5 (6)	H23A—C23—H23B	108.2
C5—C10—C19	112.9 (6)	C25—C24—C23	111.1 (9)
C9—C10—C1	111.2 (5)	C25—C24—H24A	109.4
C5—C10—C1	104.8 (6)	C23—C24—H24A	109.4
C19—C10—C1	107.6 (6)	C25—C24—H24B	109.4
C9—C11—C12	114.7 (6)	C23—C24—H24B	109.4
C9—C11—H11A	108.6	H24A—C24—H24B	108.0
C12—C11—H11A	108.6	C24—C25—C26	109.1 (10)
C9—C11—H11B	108.6	C24—C25—C27	114.5 (12)
C12—C11—H11B	108.6	C26—C25—C27	109.9 (10)
H11A—C11—H11B	107.6	C24—C25—H25	107.7
O2—C12—C11	121.2 (8)	C26—C25—H25	107.7
O2—C12—C13	123.7 (8)	C27—C25—H25	107.7
C11—C12—C13	114.9 (7)	O4—C26—C25	110.9 (9)
C12—C13—C14	105.4 (5)	O4—C26—H26A	109.5
C12—C13—C18	107.3 (6)	C25—C26—H26A	109.5
C14—C13—C18	113.6 (6)	O4—C26—H26B	109.5
C12—C13—C17	113.9 (6)	C25—C26—H26B	109.5
C14—C13—C17	103.4 (6)	H26A—C26—H26B	108.0
C18—C13—C17	113.2 (5)	C25—C27—H27A	109.5
C8—C14—C13	117.2 (6)	C25—C27—H27B	109.5
C8—C14—C15	122.1 (6)	H27A—C27—H27B	109.5
C13—C14—C15	100.1 (5)	C25—C27—H27C	109.5
C8—C14—H14	105.3	H27A—C27—H27C	109.5
C13—C14—H14	105.3	H27B—C27—H27C	109.5
			
C10—C1—C2—C3	−47.3 (10)	C18—C13—C14—C8	59.6 (8)
C1—C2—C3—O1	−143.0 (9)	C17—C13—C14—C8	−177.3 (6)
C1—C2—C3—C4	38.6 (12)	C12—C13—C14—C15	168.1 (6)
O1—C3—C4—C5	138.9 (9)	C18—C13—C14—C15	−74.7 (7)
C2—C3—C4—C5	−42.7 (11)	C17—C13—C14—C15	48.4 (7)
C3—C4—C5—C6	179.8 (7)	C8—C14—C15—C16	−173.1 (7)
C3—C4—C5—C10	55.3 (10)	C13—C14—C15—C16	−41.8 (8)
C4—C5—C6—C7	176.0 (7)	C22—O3—C16—C15	−153.1 (7)
C10—C5—C6—C7	−57.5 (8)	C22—O3—C16—C17	−37.9 (8)
C5—C6—C7—C8	54.8 (9)	C14—C15—C16—O3	132.2 (7)
C6—C7—C8—C14	−175.6 (6)	C14—C15—C16—C17	20.1 (8)
C6—C7—C8—C9	−51.3 (10)	O3—C16—C17—C20	19.6 (8)
C14—C8—C9—C11	−46.8 (8)	C15—C16—C17—C20	135.7 (8)
C7—C8—C9—C11	−172.5 (7)	O3—C16—C17—C13	−106.9 (6)
C14—C8—C9—C10	178.2 (6)	C15—C16—C17—C13	9.2 (7)
C7—C8—C9—C10	52.4 (9)	C12—C13—C17—C20	96.2 (8)
C8—C9—C10—C5	−54.8 (7)	C14—C13—C17—C20	−150.1 (6)
C11—C9—C10—C5	170.8 (6)	C18—C13—C17—C20	−26.7 (9)
C8—C9—C10—C19	70.3 (8)	C12—C13—C17—C16	−149.3 (6)
C11—C9—C10—C19	−64.1 (8)	C14—C13—C17—C16	−35.5 (6)
C8—C9—C10—C1	−170.2 (6)	C18—C13—C17—C16	87.8 (7)
C11—C9—C10—C1	55.4 (8)	C16—C17—C20—C21	132.4 (7)
C4—C5—C10—C9	−177.9 (6)	C13—C17—C20—C21	−114.9 (8)
C6—C5—C10—C9	56.5 (7)	C16—C17—C20—C22	4.1 (9)
C4—C5—C10—C19	58.5 (9)	C13—C17—C20—C22	116.8 (8)
C6—C5—C10—C19	−67.1 (8)	C16—O3—C22—O4	−72.2 (8)
C4—C5—C10—C1	−58.4 (8)	C16—O3—C22—C20	41.5 (9)
C6—C5—C10—C1	176.0 (6)	C16—O3—C22—C23	166.1 (7)
C2—C1—C10—C9	173.9 (7)	C26—O4—C22—O3	−61.1 (9)
C2—C1—C10—C5	55.4 (8)	C26—O4—C22—C20	−174.6 (6)
C2—C1—C10—C19	−64.9 (9)	C26—O4—C22—C23	59.7 (9)
C8—C9—C11—C12	45.3 (9)	C21—C20—C22—O3	−154.6 (8)
C10—C9—C11—C12	179.9 (6)	C17—C20—C22—O3	−27.6 (10)
C9—C11—C12—O2	135.2 (9)	C21—C20—C22—O4	−38.3 (10)
C9—C11—C12—C13	−49.4 (9)	C17—C20—C22—O4	88.7 (9)
O2—C12—C13—C14	−133.0 (9)	C21—C20—C22—C23	84.7 (10)
C11—C12—C13—C14	51.8 (9)	C17—C20—C22—C23	−148.4 (8)
O2—C12—C13—C18	105.7 (10)	O3—C22—C23—C24	67.5 (10)
C11—C12—C13—C18	−69.5 (8)	O4—C22—C23—C24	−53.5 (10)
O2—C12—C13—C17	−20.4 (11)	C20—C22—C23—C24	−173.8 (8)
C11—C12—C13—C17	164.4 (6)	C22—C23—C24—C25	52.5 (12)
C7—C8—C14—C13	−177.9 (6)	C23—C24—C25—C26	−54.3 (12)
C9—C8—C14—C13	56.3 (8)	C23—C24—C25—C27	−178.0 (7)
C7—C8—C14—C15	−54.1 (9)	C22—O4—C26—C25	−62.2 (11)
C9—C8—C14—C15	−180.0 (7)	C24—C25—C26—O4	59.1 (12)
C12—C13—C14—C8	−57.6 (8)	C27—C25—C26—O4	−174.6 (10)
